# An exonic insertion within *Tex14 *gene causes spermatogenic arrest in pigs

**DOI:** 10.1186/1471-2164-12-591

**Published:** 2011-12-02

**Authors:** Anu Sironen, Pekka Uimari, Heli Venhoranta, Magnus Andersson, Johanna Vilkki

**Affiliations:** 1 Agrifood Research Finland, MTT, Biotechnology and Food Research, Genomics, FI-36100 Jokioinen, Finland; 2 University of Helsinki, Department of Clinical Veterinary Sciences, Saarentaus, Finland

## Abstract

**Background:**

Male infertility is an increasing problem in all domestic species including man. Localization and identification of genes involved in defects causing male infertility provide valuable information of specific events in sperm development. Sperm development is a complex process, where diploid spermatogonia develop into haploid, highly specialized spermatozoa. Correct expression and function of various genes and their protein products are required for production of fertile sperm. We have identified an infertility defect in Finnish Yorkshire boars caused by spermatogenic arrest. The aim of this study was to locate the disease associated region using genome wide screen with the PorcineSNP60 Beadchip and identify the causal mutation by candidate gene approach.

**Results:**

In the Finnish Yorkshire pig population the spermatogenic arrest (SA) defect appears to be of genetic origin and causes severe degeneration of germ cells and total absence of spermatozoa. Genome wide scan with the PorcineSNP60 Beadchip localized the SA defect to porcine chromosome 12 in a 2 Mbp region. Sequencing of a candidate gene *Tex14 *revealed a 51 bp insertion within exon 27, which caused differential splicing of the exon and created a premature translation stop codon. The expression of *Tex14 *was markedly down regulated in the testis of a SA affected boar compared to control boars and no protein product was identified by Western blotting. The SA insertion sequence was also found within intron 27 in all analyzed animals, thus the insertion appears to be a possible duplication event.

**Conclusion:**

In this study we report the identification of a causal mutation for infertility caused by spermatogenic arrest at an early meiotic phase. Our results highlight the role of TEX14 specifically in spermatogenesis and the importance of specific genomic remodeling events as causes for inherited defects.

## Background

Male infertility is a significant problem in all mammalian species [1]. In humans, it is estimated that 15% of couples are infertile and in one third of these cases infertility can be attributed solely to the male partner [2]. In productive livestock the economic losses due to reproductive inefficiency in males can be substantial, particularly when infertility affects a genetically superior individual [3]. Although some instances of male factor infertility can be explained by infections, environmental causes, immunological or hormonal deficiencies, many are caused by genetic factors. Problems with the production and maturation of spermatozoa are the most common causes of male infertility resulting in a low sperm count, morphologically abnormal spermatozoa or reduced sperm motility [4-6]. Despite efforts to reveal the genes involved in spermatogenesis and their functions, little is known about the underlying causes of male infertility. Therefore, the localization and identification of mutations affecting specifically the spermatogenesis provide invaluable information for research on the causes of male infertility.

Mammalian spermatogenesis is a complex process, where diploid spermatogonia develop into haploid, highly specialized spermatozoa. Spermatogenesis occurs in the seminiferous tubules of the testis and can be divided in proliferative phase, meiotic phase and differentiation (spermiogenesis) [7]. During the proliferative phase spermatogonia go through several mitotic divisions. The final mitotic division of differentiated spermatogonia gives rise to the primary spermatocytes. Meiosis of primary spermatocytes leads to the production of secondary spermatocytes after the first meiotic division, while haploid round spermatids are formed following the second meiotic division. After meiosis, spermatids are connected with cytoplasmic bridges sharing transcripts and proteins [8]. In spermiogenesis the nucleus of the germ cell is remodeled and the acrosome and sperm tail are formed. Finally, mature spermatozoa are released into the lumen of seminiferous epithelium and transported to the epididymis for further maturation. During the whole process, the expression and the interactions between various genes and their protein products are required and regulated in an ordered manner. Some of these genes or their alternative transcripts are specifically expressed in the testis. Identification of these genes and their roles is important in understanding the mechanism of spermatogenesis.

Spermatogenic arrest at various stages of spermatogenesis is known to cause infertility in several mammalian species [9-11], but only recently genes and interaction networks behind these defects have been under investigation. Different mouse models have revealed several genes associated with male infertility caused by spermatogenic arrest [12-15], but the full understanding of events leading to spermatogenic arrest at specific phases requires further studies. Within the Finnish Yorkshire population several boars with azoospermia were identified during years 1995-2008. In this study we report an infertility defect in the Finnish Yorkshire boars caused by spermatogenic arrest (SA) in early meiotic cells. The aim of the study was to identify the mutation causing the SA defect and reveal the role of the affected gene in spermatogenesis. The disease associated region was localized to porcine chromosome 12 within a 1.9 Mbp region and a promising candidate gene, *Testis expressed 14 *(*Tex14*), was identified. Sequencing of the *Tex14 *mRNA showed a SA specific insertion in exon 27, which resulted in a premature translation stop codon and decreased expression of the TEX14 mRNA and protein products specifically in the testis.

## Results

### Spermatogenic arrest in Finnish Yorkshire boars results in severe degeneration of germ cells

**Figure 1:**
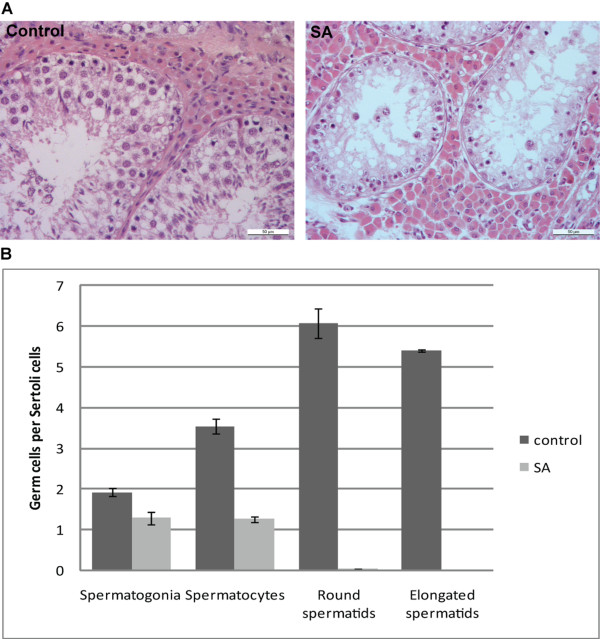
**The spermatogenesis is markedly affected in SA boars**. A. In the testis cross sections of SA affected boars no spermiogenic cells are present and increased vacuolization can be seen compared to the control testis. B. Clear reduction in the amount of spermatogonia and spermatocytes in SA affected animals can be seen compared to control animals. No spermatids are present in the testis of SA boars. The number of germ cells is compared to the amount of Sertoli cells.

The infertility of SA affected boars was first identified in boar stations. The testicular size appeared to be approximately half of the normal size in affected boars and microscopical examination of the ejaculate revealed total absence of spermatozoa [16]. Further histological examination of the SA affected testis sections showed markedly reduced number of late meiotic cells and absence of postmeiotic cells (Figure 1, [16]). Furthermore, affected testes displayed severe degeneration of germ cells and vacuolization probably due to increased germ cell apoptosis (Figure 1).

### Genome wide association analysis localized the SA defect in porcine chromosome 12

**Figure 2:**
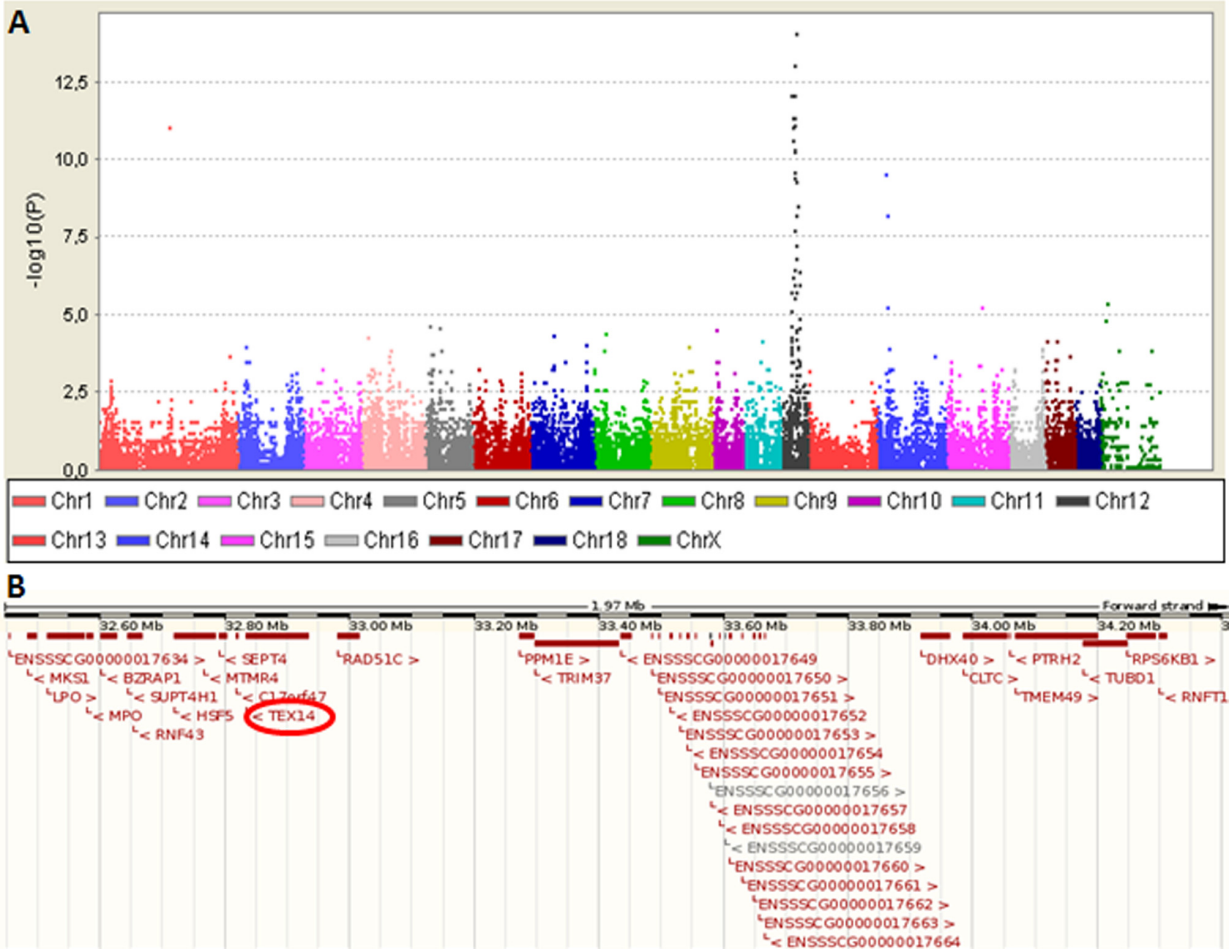
**The SA associated region in the Finnish Yorkshire was identified in chromosome 12**. A. Manhattan plot showing the P-values (-log10(P) on the y-axis) for different chromosomes using a recessive mode of inheritance-model based on 9 cases and 21 controls. B. The SA associated homozygous region between 32.5-34.4 Mbp contains a promising candidate gene *Testis expressed 14 *(*Tex14*).

Call rate (the proportion of successfully genotyped SNPs over all SNPs on the chip) was over 95% for all genotyped samples in this study. In the initial genome wide screen (GWS), 27,510 SNPs had minor allele frequency (MAF) > 0. The Manhattan plot of P-values of these SNPs is shown in Figure 2A. In the initial GWS 45 SNPs had P-value less than 1.81E-06 that corresponds to Bonferroni corrected overall P-value < 0.05 (Additional file 1, Table S1). All the significant SNPs were located in a 10 Mbp long region (position: 23.9 - 34.1 Mb, Sscrofa9, http://www.ensembl.org) on chromosome 12 except one SNP on chromosome 1 (ALGA0065844). This single SNP is most likely a false positive finding and was ignored in the later analysis.

When a larger control group was used in GWS only two SNPs (ALGA0066210 and ALGA0066216) supported a fully penetrant recessive mode of inheritance (Additional file 1, Table S1). All SA affected boars, but none of the controls, were homozygous for alleles C and A in ALGA0066210 and ALGA0066216, respectively. Additionally, all SA affected boars had two copies of the same haplotype containing 29 SNPs and all controls that were heterozygous for ALGA0066210 and ALGA0066216 had one copy of that haplotype (haplotype 1 in Table 1). Thus, there appears to be a single origin for the SA causal mutation.

**Table 1:** The most common haplotypes at the region 32.5 - 34.4 Mbp on chromosome 12 and their frequencies among cases and controls.

		Haplotypes							
SNP	Position	1	2	3	4	5	6	7	8
DIAS0000466	32486728	A	A	G	A	A	A	A	A
DRGA0011732	32552621	C	C	A	C	C	C	C	C
MARC0040388	32564534	G	A	G	G	G	G	A	A
ALGA0066208	32594630	G	A	G	A	A	A	A	A
**ALGA0066210**	**32620047**	**C**	**A**	**A**	**A**	**A**	**A**	**A**	**A**
ASGA0054360	32676377	A	G	G	G	G	G	A	G
ALGA0066214	32706622	A	C	C	C	C	A	A	A
ASGA0054362	32729466	A	A	A	A	A	G	G	G
MARC0016326	32762378	A	G	G	G	G	A	A	A
**ALGA0066216**	**32843547**	**A**	**G**	**G**	**G**	**G**	**G**	**G**	**G**
ALGA0116573	32936852	A	A	G	A	G	A	A	A
ALGA0066218	33179967	G	G	A	G	A	G	G	G
ALGA0066217	33236507	G	A	G	G	G	A	G	A
ASGA0097668	33738117	A	A	A	G	A	G	A	A
ASGA0103400	33776732	A	A	A	A	A	G	A	A
ALGA0066221	33825748	A	G	A	A	A	A	G	A
ALGA0066222	33852536	G	A	A	G	G	A	A	A
MARC0039239	33887438	C	C	A	C	C	A	C	C
DRGA0011741	33924664	C	A	A	C	A	A	A	A
DRGA0011742	33965584	A	A	A	A	A	A	A	G
INRA0038984	34016194	A	C	A	C	A	C	C	A
ALGA0066230	34081571	A	G	G	G	A	G	G	G
H3GA0034268	34115708	C	C	A	A	C	A	C	A
ALGA0066234	34129776	G	A	G	G	A	G	A	G
MARC0084960	34150307	G	G	A	A	G	A	G	A
H3GA0034269	34183841	G	A	A	G	G	A	A	A
ASGA0054380	34233636	G	G	A	A	A	G	G	G
H3GA0034274	34343301	C	C	C	C	A	C	C	A
ALGA0066247	34362626	G	G	G	G	G	A	G	A

Cases		18	0	0	0	0	0	0	0
Controls		25	146	129	87	78	49	47	47

Haplotype 1 is the longest haplotype that is shared by all cases in this chromosome region. The SNPs that fulfil recessive mode of inheritance in the large data set are marked in bold face.

The SA associated haplotype covers a 2 Mbp region in SSC 12, which contains a large number of annotated genes (Figure 2B). Within these genes a promising candidate gene for the spermatogenic arrest, *Tex14*, was found at position 32834226-32934785 bp. *Tex14 *has been shown to be crucial for successful spermatogenesis having a role in converting midbodies into stable intercellular bridges [17]. In addition, *Tex14 *knockout mice have a similar spermatogenic phenotype [15] as was seen in SA affected boars. One of the SNPs with a strong association with the SA defect, ALGA0066216, also resides within the *Tex14 *genomic sequence.

### SA defect in the Finnish Yorkshire population is caused by an exonic insertion

**Figure 3:**
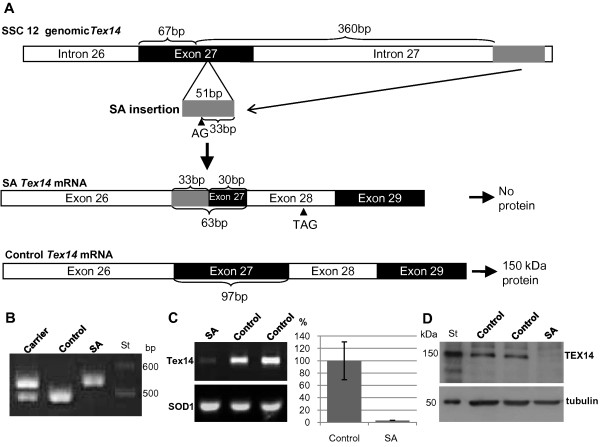
**The SA defect is caused by an insertion within exon 27 of *Tex14***. A. A genomic insertion of 51 bp originating from *Tex14 *intron 27 (grey bar) has been duplicated into exon 27. This duplication carries an additional splicing site (AG) 18 bp from the duplication start site. In the mRNA of SA affected testis the *Tex14 *exon 27 has been replaced by 33 nt of the direct duplication and 30 nt of the 3' end of the exon 27. Thus, the 67 bp of the 5' end of the exon 27 is absent in the *Tex14 *mRNA of SA affected boars. The aberrant splicing in the mRNA creates a premature translation stop codon (TAG) in the exon 28. B. The genomic insertion of 51 bp can be detected on an agarose gel with PCR primers adjacent to the insertion. C. *Tex14 *expression (exons 17-19) is markedly lower in the SA affected testis compare to control boars. The expression difference was quantified with qPCR (P < 0.001) and is presented as percentage of the control testis expression. *SOD1 *gene was used as a loading control. D. TEX14 protein expression in the SA affected and control testis was evaluated by western blotting. TEX14 appeared to be absent in the SA affected testis. α-tubulin was used as a loading control.

The predicted pig *Tex14 *transcript [EMBL: ENSSSCT00000019208] contains 4496 nt and 34 exons. Sequencing of the control testis mRNA of *Tex14 *[GenBank: JN638886] showed a transcript including 4194 nt. Sequence alignment with the Sequencer software (Gene Codes Corporation) showed differences in exon content between the pig *Tex14 *database sequence and our results. The testis mRNA starts at exon 2 and one additional exon was present after exon 13. In addition, the sequence of exon 15 was different in the EMBL transcript and in the testis mRNA and exon 16 was missing in our data. The translation stop codon locates after position 4191 nt in our testis transcript, thus the pig TEX14 protein sequence in the testis contains 1397 aa. The protein sequence contains ankyrin repeat region at position 1-99 (E-value 5.7e-16) and PKc_like-domain at position 154 - 466 aa (E-value 1.9e-14). Sequencing of a control and SA affected boar samples revealed several polymorphisms within the coding region of *Tex14 *(Table 2). Five of these polymorphisms lead to amino acid changes possibly affecting the protein structure and function. However, the most likely cause for the SA defect was found in exon 27. In the mRNA sequence of all SA affected boars a 67 nt deletion (del) and a 33 nt insertion (ins) was found in the exon 27. This del/ins resulted in a premature translation stop codon at aa 1287 in the SA affected mRNA (Figure 3A).

**Table 2:** SA associated polymorphisms detected within the *Tex14 *mRNA.

Position nt	Control	SA affected	Protein	Position aa
388	A	G	S - > G	131
461	G	C		
1140	G	A		
1419		GGC	+G	475
1677	C	A		
1755	T	C		
1941	A	G		
1994	C	T	S - > L	665
2139	C	T		
2228	G	A	R - > H	743
2466	A	G		
2511	T	G		
3307	G	A	G - > S	1103
3844		66 bp deletion +33 bp insertion	KRLPA- > RETS stop	1283-1287
4061	G	A	N - > S	1354

The effect of the polymorphism on protein sequence and position in control transcript [GenBank: JN638886] and corresponding protein sequence are indicated.

The identified region containing the del/ins polymorphism in the *Tex14 *mRNA was further analysed in the genomic DNA of all SA affected boars and a group of control animals (n = 40). All affected boars had a homozygous 51 bp insertion within the genomic sequence of *Tex14 *exon 27. The insertion creates a novel exon/intron splicing acceptor site causing differential splicing of this exon in affected animals, thus creating the identified del/ins in the *Tex14 *mRNA (Figure 3A). In control boars five heterozygous animals were identified and in 35 analyzed boars the exonic insertion was absent. In addition, DNA from two sires of SA affected boars was available and both of these boars were heterozygous for the insertion.

In order to identify the origin of the 51 bp insertion, a Blast search against the pig genome build 9 was done. The insertion sequence was found in the *Tex14 *intron 27. The presence of this intronic sequence in SA affected animals was confirmed by sequencing. Thus, the causal mutation for spermatogenic arrest in the Finnish Yorkshire population appears to be a duplication of a 51 bp sequence within *Tex14 *gene (Figure 3A).

### Tex14 expression is decreased in the testes of SA affected boars

**Figure 4:**
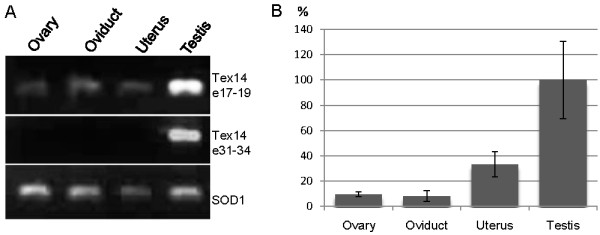
**Expression of *Tex14 *in female reproductive tracts**. A. The expression of two *Tex14 *fragments up- and downstream of the SA duplication site was investigated in the ovary, oviduct and uterus of control animals. *Tex14 *exons 17-19 were slightly expressed in all examined female tissues, but the expression appeared markedly lower than in the testis. Exons 31-43 of *Tex14 *showed no expression in any of the female reproductive tissues confirming the significantly lower expression of *Tex14 *in the ovary, oviduct and uterus compared to the testis. B. The quantity of *Tex14 *exons 17-19 expression in the ovary, oviduct and uterus in comparison to the testis was analysed with qPCR and is presented as percentage of the testis expression. *Tex14 *expression was significantly (P < 0.001) lower in all examined tissues compared to the testis.

The expression of *Tex14 *mRNA was examined by RT-PCR on agarose gels and qPCR. Both methods indicated clear reduction in *Tex14 *expression in the SA affected testis (Figure 3C). Furthermore, TEX14 protein product was absent in the testis of a SA affected boar (Figure 3D). TEX14 has a role in the formation of embryonic intercellular bridges, which are formed during the male and female gametogenesis [18] indicating that down regulation of *Tex14 *expression may also affect the female fertility. Since no samples from homozygous sows for the *Tex14 *insertion were available, we investigated the expression of *Tex14 *in the ovary, oviduct and uterus of control sows. *Tex14 *exons 17-19 were slightly expressed also in all female tissues, but no expression of exons 31-34 was detected in the agarose gel (Figure 4A). The *Tex14 *expression was also quantified with qPCR, which indicated a markedly lower expression in the female reproductive organs compared to the testis (Figure 4B). In addition, no other symptoms have been detected in SA boars and no problems in reproductive performance in sows have been reported indicating that the SA defect affects only male fertility. The SA phenotype and low *Tex14 *expression in the ovary, oviduct and uterus indicate a minor role for TEX14 in these tissues and therefore the premature translation stop codon may not have a major effect on female fertility. These data highlight the role of TEX14 and the formation of intercellular bridges specifically in spermatogenesis.

### Marker and gene assisted selection available for selection against the spermatogenic arrest defect

Gene assisted selection based on the causal mutation of the SA defect provides a 100% accurate DNA test against the defect. PCR amplification with sequence specific primers flanking the 51 bp insertion in the genomic sequence of *Tex14 *gene can be used for gene assisted selection against the SA defect in the Yorkshire population. The size difference of the PCR fragment can be detected on agarose gel (Figure 3B) and the disease status defined as SA affected, carrier or control. The fragment size for the allele without the insertion is 496 bp (control) and including the insertion is 547 bp (affected).

In order to avoid additional gene test we also evaluated the usability of associated SNPs for marker assisted selection. Heterozygous boars (n = 17) for SNPs ALGA0066210 and ASGA0054360 from Illumina beadchip genotyping were selected for sequencing with primers flanking the *Tex14 *insertion. Five boars were heterozygous for both SNPs and appeared to be SA carriers based on the *Tex14 *insertion. These boars were also carriers of the haplotype 1 (Table 1). None of the boars (n = 12) heterozygous only for one SNP were SA carriers. SA associated alleles were C and A for SNPs ALGA0066210 and ASGA0054360, respectively. Thus, the haplotype of these markers appears to be a reliable indicator for the presence of the SA insertion at least in the Finnish Yorkshire population.

## Discussion

The SA affected boars show almost no late meiotic and postmeiotic germ cells and reduced amount of spermatogonia. This phenotype is very similar to *Tex14 *knockout mice phenotype and therefore the search for causal mutation was focused on *Tex14 *gene. In *Tex14 *knockout mice, initially markedly reduced numbers of late meiotic and post meiotic cells are detected and in older animals more severe phenotype with decrease in all germ cell numbers is present [15]. In addition, the vacuolization is increased in older animals in the mouse and was evident also in the pig. In mice, as spermatogonia divide and differentiate, TEX14 together with other midbody proteins form intercellular bridges, a stable structure formed by mitotic and meiotic divisions and maintained until formation of spermatozoa. TEX14 interaction with CEP55 has been shown to be critical for subverting abscission toward a stable intercellular bridge [19].

In *Tex14 *knockout male mice, germ cells continue to form midbodies during telophase of cytokinesis, but the midbody is transient, as in somatic cells, and no intercellular bridges are formed [17]. Thus, *Tex14 *knockout mice completely lack intercellular bridges, and spermatogenesis fails before the first meiotic division resulting in male infertility [15]. In Finnish Yorkshire boars the spermatogenic arrest at first meiotic division appears also to be caused by a mutation in the *Tex14 *gene. Although several polymorphisms were detected within the *Tex14 *coding frame, a 51 bp insertion in exon 27 appears to be the obvious cause for the defect. This insertion results in differential splicing of exon 27, which creates a premature translation stop codon after position 3858 nt in the SA affected *Tex14 *transcript. The expression of all examined *Tex14 *fragments were markedly down regulated in the SA affected boar and the protein product of 150 kDa was absent. The probable cause for the down regulation of the *Tex14 *mRNA is the nonsense mediated degradation of the incorrectly spliced mRNA, but we have not entirely excluded the possibility of down regulation of mRNA transcription or changes in cytoplasmic mRNA stability. The sequencing of the promoter region (152 bp upstream of the transcription initiation site) did not show any SA associated mutations indicating that the transcription is not affected by mutations in the promoter region, but regulatory regions further upstream have not been investigated. However, the protein product of TEX14 appears to be absent in the SA affected testis highlighting the role of this gene in development of the SA defect.

In the Finnish Yorkshire pigs no effect on female reproduction has been reported and no other symptoms except infertility in boars were detected. Thus, the insertion within *Tex14 *appears to only affect spermatogenesis. This has also been seen in mice. In *Tex14 *knockout females no significant difference in the number of germ cells at prenatal day 11.5 was detected and litter size was only slightly affected, although the intercellular bridges were missing [18]. In this study we have also analyzed the expression of *Tex14 *in the testis, ovary, oviduct and uterus of control pigs. *Tex14 *appears to be highly expressed in the testis and substantially lower expression or no expression was detected in the female reproductive organs indicating a more prominent role of TEX14 in the testis. This result is consistent with studies in the mouse showing that TEX14 is not essential for postnatal oocyte growth and ovarian folliculogenesis [18].

The ancestral SA insertion sequence locates in the pig genome within *Tex14 *intron 27 360 bp downstream from the SA insertion site. All affected boars also have the original sequence in intron 27, thus the disease causing fragment appears to be copied in the same orientation to exon 27. The SA insertion region was not identified as an interspersed repeat or a low complexity DNA sequence, thus it appears to be a unique duplication event in the porcine genome. In the human, indels cause multiple genetic diseases [20] and are a source of natural intra- and interspecific genetic variation [21-24]. Short unique sequences are being actively duplicated in mammalian genomes and are often separated by some distance [25]. It has been postulated that distantly located duplicates are generated by direct tandem duplications, and separated apart by later insertions [26]. However, the SA insertion into an existing target sequence was created with minimal collateral damage to the target. This separated tandem duplication may have arisen by recombination after duplication event, since duplications undergo high frequencies of recombination and are consequently unstable [25]. Duplications have been shown to attribute from replication slippage errors [27,28], but also the significance of recombination to the formation of small indels, in particular insertions, has been demonstrated [29,30]. However, these mechanisms require an existing duplicate in the DNA sequence [28]. In the pig, within the region containing the SA insertion sequence and original sequence within *Tex14 *intron 27 no duplication sequences were present in the wild type animals. One possible mechanism for the SA duplication event could also be the imperfect repair of DNA double-strand breaks by classical nonhomologous end joining (NHEJ) [31-33]. NHEJ requires only short microhomologies of 1-4 bp and can even ligate overhangs without homologies [28,34].

The identification of the insertion within *Tex14 *exon 27 enables the gene assisted selection against the defect. Although the SA carrier status is fairly easy to determine on an agarose gel, we further examined the possibility to use SNPs on Illumina PorcineSNP60 beadchip for identification of the SA carriers and affected animals. Two SNPs (ALGA0066210 and ASGA0054360) were strongly associated with the SA defect and a haplotype of these SNPs appears to be a usable marker for marker assisted selection against the defect at least within the Finnish Yorkshire population.

*Tex14 *gene appears to be crucial for succesful spermatogenesis in the mouse and pig. The functional studies revealing the role of TEX14 in the intercellular bridges between developing male germ cells indicate the importance of this gene in sperm production across species. A statistically significant difference in *Tex14 *gene expression has also been identified in azoospermic men [35]. However, no statistically significant associations between *Tex14 *SNPs and azoospermia have been identified in this study or in other genome wide association analyses of azoospermic men [36]. The lack of statistically significant associations may be partly due to the heterogenity of azoospermic cases and low frequency of *Tex14 *mutations. Genome wide association analyses of heterogenic samples require high number of cases and controls. Thus, the low amount of samples hinders the analyses in these studies and larger genome-wide association studies are required in order to identify novel SNPs associated with azoospermia. However, *Tex14 *is a good candidate gene for causal mutations in azoospermic men.

## Conclusions

In this study we report a specific infertility defect in Yorkshire pigs. The infertility is caused by spermatogenic arrest in early meiotic sperm cells resulting in absence of spermatocytes and spermatids. The genome wide scan located the disease associated area within 2 Mbp region in porcine chromosome 12. Sequencing of a candidate gene, *Tex14*, revealed several polymorphisms within the coding sequence. The most likely causal mutation was identified within exon 27 resulting in premature stop codon in the mRNA sequence. In addition, the expression of TEX14 mRNA and protein products was markedly down regulated in the testis of a SA affected boar compared to control animals. The genomic mutation appeared to be a 51 bp insertion, which originates from *Tex14 *intron 27. Thus, the SA insertion is probably an original duplication event separated by recombination.

## Methods

### Animal material and genotyping

In the initial genome wide scan the data set included nine SA affected Finnish Yorkshire boars (cases) and 21 control boars. The larger follow up study included additional 318 control boars that have been used in artificial insemination. DNA was extracted from semen following phenol/chloroform extraction. For each sample, 20 μl of extracted DNA with target DNA concentrations of 100 ng/μl in TE-buffer was provided for the Institute for Molecular Medicine Finland (FIMM) where genotyping was conducted using the PorcineSNP60 BeadChip (Illumina Ltd, San Diego, USA) and the protocol provided by the manufacturer.

The gene test for the *Tex14 *insertion within exon 27 was performed using genomic DNA and gene specific primers (forward GTAAGACTGGCATGATGTAACACAGAA and reverse ACTCAGGGTATCTTTCTGGAGTTCT).

### PCR amplification and DNA sequencing

PCR amplification of *Tex14 *using SA affected and control cDNA extracted from the testis or genomic DNA as a template was performed with gene specific primers. The PCR amplicons were purified using ExoSAP-IT™ (Amersham Biosciences) and sequenced in both directions with the same primers used in the amplification procedures. Sequencing was performed on MegaBace 500 capillary DNA sequencer (Amersham Biosciences) using DYEnamic ET Terminator Kits with Thermo Sequenase™ II DNA Polymerase (Amersham Biosciences).

### Gene expression

For analysis of candidate gene expression, samples of the oviduct, ovary and uterus from two normal sows and testicular tissue from two normal and one SA affected boar were collected and stored in RNAlater buffer (Qiagen). Total RNA purification was performed with RNeasy Protect Mini and Midi kits (Qiagen). Total RNA was reverse transcribed (RT-PCR) with random primers and an RNA PCR kit (ImProm-II Reverse Transcription System, Promega, http://www.promega.com) according to the manufacturer's instructions and amplified using gene specific primers. Expression of *Tex14 *exons 17-19 and 31-34 was assessed first by gel electrophoresis and quantified thereafter by quantitative PCR (qPCR). Control reactions were performed with *SOD1 *gene.

qPCR was performed with an ABI 7000 Sequence Detection System in 96-well microtiter plates using Absolute qPCR SYBR Green ROX Mix (ABgene). Amplification by qPCR contained 12.5 μl of Absolute qPCR SYBR Green Mix, 50 ng of cDNA, and 70 nM of each primer in a final volume of 25 μl. Amplifications were initiated with a 15-min enzyme activation at 95°C followed by 40 cycles of denaturation at 95°C for 15 s, primer annealing at 60°C for 30 s, and extension at 72°C for 30 s. All samples were amplified in triplicate, and the mean value was used for further calculations. Each run comprised of two unaffected samples of the testis, ovary, oviduct and uterus, one SA affected testis sample and a negative control sample in triplicate. A standard curve for each primer pair was produced by serially diluting control testis cDNA. Quantities of specific mRNA in the sample were measured according to the corresponding gene-specific standard curve. Raw data were analyzed with the sequence detection software (Applied Biosystems) and relative quantitation was performed within GeneEx software (MultiD Analyses AB). Ratios between the target and reference gene were calculated by using the mean of these measurements. Specificity of qPCR products was determined by a melting curve analysis. No primer-dimer formations were detected during the application of 40 real-time PCR amplification cycles.

### Protein detection

Tissue samples were homogenized in lysis buffer (50 mM Tris-HCl [pH 8.0], 170 mM NaCl, 5 mM ethylenediaminetetra-acetic acid, 1 mM dithiothreitol, 1% NP-40, 0.5% sodium deoxycholate, 0.05% SDS and protease inhibitors [Complete mini; Roche Diagnostics GmbH]) and quantified using Bradford protein assay reagent [37]. Samples were separated under denaturing conditions by 8% SDS-PAGE and electroblotted to polyvinylidene fluoride membrane. Nonspecific sites were blocked with 5% nonfat dry milk in 0.3% Tween-20 in PBS for 1 h at room temperature, and the membrane was incubated overnight with anti-TEX14 (1:500, Sigma-Aldrich), or anti-alpha tubulin (1:1000; NeoMarkers) antibody. Antigen-antibody complexes were detected by incubation of the membranes with the anti-rabbit or anti-mouse secondary antibody (horseradish peroxidase-conjugated; Bio-Rad) for 2 h at room temperature. The bound secondary antibodies were located with the Immun-Star WesternC Chemiluminescent Kit (Bio-Rad) according to the manufacturer's instructions and exposed to a film thereafter.

### Statistical analysis

Each SNP was tested for a recessive mode of inheritance where the frequency of recessive homozygotes was compared to the frequency of heterozygotes and the other homozygotes between SA affected (cases) and non-affected boars (controls). The recessive mode of inheritance -model was chosen, because the SA affected boars were related and the frequency of the defect is low in the population. When several thousand SNPs are tested, the probability of false positive findings is high. In order to avoid false positive findings only SNPs that remained significant after Bonferroni correction for number of informative SNPs were considered as statistically significant (P < 1.81E-06). Software package Plink [38] was used for the association test. Manhattan and linkage disequilibrium plots and the most plausible haplotypes were produced with Haploview software [39].

## Authors' contributions

AS carried out the molecular genetics studies, sequence alignments and drafted the manuscript. PU performed the statistical analysis and participated in drafting the manuscript. HV participated in the sequence analysis. MA participated in the design and coordination of the study. JV participated in the design and helped to draft the manuscript. All authors read and approved the final manuscript.

## Supplementary Material

Additional file 1 **Significant SNPs (after Bonferroni correction) on chromosome 12 based on a data set with 9 cases and 21 controls (P-value small set) and corresponding P-values from the larger data set with 339 controls (P-value large set)**. The SNPs that fulfil recessive mode of inheritance in the large data set are marked in bold face. Click here for file
