# Supplementary material for: An exonic insertion within Tex14 gene causes spermatogenic arrest in pigs
Source: BMC Genomics. 2011 Dec 2;12:591. doi: 10.1186/1471-2164-12-591 (PMC3248578; doi:10.1186/1471-2164-12-591)
Supplement: Additional file 1 — Significant SNPs (after Bonferroni correction) on chromosome 12 based on a data set with 9 cases and 21 controls (P-value small set) and corresponding P-values from the larger data set with 339 controls (P-value large set). The SNPs that fulfil recessive mode of inheritance in the large data set are marked in bold face. [file 1471-2164-12-591-S1.DOCX]

**Additional file 1: table S1.** Significant SNPs (after Bonferroni correction) on chromosome 12 based on a data set with 9 cases and 21 controls (P-value small set) and corresponding P-values from the larger data set with 339 controls (P-value large set). The SNPs that fulfil recessive model of inheritance in the large data set are marked in bold face.

| SNP | Position | Allele 1 | Allele 2 | Cases^a^ | Controls  small set  small set^a^ | P-value  small set | Controls  large set^a^ | P-value  large set |
| --- | --- | --- | --- | --- | --- | --- | --- | --- |
| MARC0096817 | 23887163 | G | A | 8/0/1 | 0/0/21 | 4.53E-07 | 1/41/288 | 8.33E-60 |
| MARC0063635 | 23913026 | A | G | 8/0/1 | 0/0/21 | 4.53E-07 | 0/38/299 | 1.18E-68 |
| CASI0006966 | 25026706 | A | C | 8/0/1 | 0/1/20 | 4.53E-07 | 4/96/238 | 7.71E-46 |
| ALGA0108989 | 25033240 | A | G | 9/0/0 | 0/3/18 | 4.32E-08 | 26/135/176 | 1.29E-19 |
| DIAS0001577 | 25075214 | G | A | 9/0/0 | 0/4/17 | 4.32E-08 | 59/152/127 | 7.41E-10 |
| MARC0050837 | 25172741 | G | A | 8/0/1 | 0/0/21 | 4.53E-07 | 0/32/307 | 4.82E-69 |
| ALGA0113322 | 25244680 | A | G | 8/0/1 | 0/0/21 | 4.53E-07 | 3/63/273 | 3.62E-50 |
| ALGA0105569 | 25320047 | C | A | 9/0/0 | 0/3/18 | 4.32E-08 | 27/137/172 | 5.28E-19 |
| MARC0054687 | 25734376 | A | G | 8/0/1 | 0/1/20 | 4.53E-07 | 0/82/257 | 4.82E-69 |
| ALGA0065826 | 25841230 | A | C | 8/0/1 | 0/1/20 | 4.53E-07 | 3/91/244 | 5.01E-50 |
| ALGA0065830 | 25864248 | A | G | 8/0/1 | 0/0/21 | 4.53E-07 | 0/34/305 | 4.82E-69 |
| CASI0009146 | 25909369 | A | G | 8/0/1 | 0/1/20 | 4.53E-07 | 5/107/227 | 2.05E-42 |
| MARC0039241 | 26120823 | G | A | 8/0/1 | 0/0/21 | 4.53E-07 | 3/61/275 | 3.62E-50 |
| ALGA0116215 | 26146570 | A | G | 8/0/1 | 0/0/21 | 4.53E-07 | 6/68/265 | 2.29E-39 |
| MARC0114449 | 26543124 | G | A | 9/0/0 | 0/3/18 | 4.32E-08 | 32/137/166 | 1.41E-16 |
| ALGA0065842 | 26572109 | A | G | 9/0/0 | 0/4/17 | 4.32E-08 | 33/142/163 | 2.58E-16 |
| MARC0083256 | 27266958 | A | G | 9/0/0 | 0/3/18 | 4.32E-08 | 44/142/152 | 8.12E-13 |
| ASGA0053947 | 27581354 | G | A | 8/0/1 | 0/0/21 | 4.53E-07 | 3/63/273 | 3.62E-50 |
| ALGA0065869 | 27948553 | G | A | 8/0/1 | 0/0/21 | 4.53E-07 | 0/30/303 | 6.99E-68 |
| MARC0044696 | 28000795 | A | C | 9/0/0 | 0/3/18 | 4.32E-08 | 34/125/177 | 7.87E-16 |
| H3GA0034030 | 28073646 | A | G | 9/0/0 | 0/3/18 | 4.32E-08 | 44/142/152 | 8.12E-13 |
| ASGA0053963 | 28213941 | G | A | 9/0/0 | 0/3/18 | 4.32E-08 | 43/141/152 | 5.36E-13 |
| DRGA0011696 | 28325555 | A | C | 9/0/0 | 0/3/18 | 4.32E-08 | 44/143/151 | 8.12E-13 |
| DRGA0011702 | 28461266 | A | G | 8/0/1 | 0/0/21 | 4.53E-07 | 1/45/289 | 1.15E-60 |
| ALGA0065904 | 28559780 | G | A | 9/0/0 | 0/3/18 | 4.32E-08 | 43/141/152 | 5.36E-13 |
| ASGA0054039 | 29702374 | A | G | 8/1/0 | 0/3/18 | 4.53E-07 | 21/122/195 | 9.13E-19 |
| ALGA0065979 | 30315234 | A | G | 8/1/0 | 0/3/18 | 4.53E-07 | 29/138/168 | 1.77E-14 |
| ALGA0065989 | 30354341 | G | A | 8/1/0 | 0/3/18 | 4.53E-07 | 27/139/170 | 2.22E-15 |
| ALGA0065995 | 30388941 | C | A | 8/1/0 | 0/3/18 | 4.53E-07 | 19/121/198 | 3.46E-20 |
| ALGA0119779 | 30506627 | A | G | 8/1/0 | 0/3/18 | 4.53E-07 | 21/120/198 | 8.06E-19 |
| MARC0054710 | 30588054 | G | A | 8/1/0 | 0/4/17 | 4.53E-07 | 32/134/171 | 1.96E-13 |
| DRGA0011720 | 30666580 | A | G | 8/1/0 | 0/4/17 | 4.53E-07 | 32/134/172 | 1.79E-13 |
| ASGA0054083 | 30687384 | G | A | 8/1/0 | 0/4/17 | 4.53E-07 | 43/136/159 | 1.90E-10 |
| MARC0030253 | 31009789 | G | A | 9/0/0 | 1/2/17 | 6.38E-07 | 6/14/30 | 2.39E-08 |
| MARC0045984 | 31070471 | G | A | 9/0/0 | 1/7/13 | 3.96E-07 | 67/155/114 | 1.07E-08 |
| ASGA0099478 | 31077658 | G | A | 8/1/0 | 0/0/21 | 4.53E-07 | 2/58/279 | 3.34E-55 |
| **ALGA0066210** | **32620047** | **C** | **A** | **9/0/0** | **0/0/21** | **4.32E-08** | **0/26/313** | **1.16E-77** |
| ASGA0054360 | 32676377 | A | G | 9/0/0 | 0/0/21 | 4.32E-08 | 2/69/268 | 1.63E-63 |
| ALGA0066214 | 32706622 | A | C | 9/0/0 | 0/8/13 | 4.32E-08 | 22/131/184 | 3.33E-22 |
| MARC0016326 | 32762378 | A | G | 9/0/0 | 0/8/13 | 4.32E-08 | 22/131/184 | 3.33E-22 |
| **ALGA0066216** | **32843547** | **A** | **G** | **9/0/0** | **0/0/21** | **4.32E-08** | **0/25/306** | **6.38E-76** |
| ASGA0099846 | 33768055 | G | A | 9/0/0 | 1/8/12 | 3.96E-07 | 2/23/28 | 2.82E-12 |
| DRGA0011741 | 33924664 | C | A | 9/0/0 | 1/4/16 | 3.96E-07 | 5/110/217 | 7.05E-49 |
| ALGA0066230 | 34081571 | A | G | 9/0/0 | 0/10/11 | 4.32E-08 | 11/104/224 | 8.07E-35 |

^a^ Genotype counts e.g. AA/AG/GG.
